# Some Sanatorium Topics

**Published:** 1912-07-13

**Authors:** 


### SOME SANATORIUM TOPICS.

There seems to be a fairly general feeling that, whatever modifications befall other sections of the Insurance Act provisions, that part of it relating to "sanatorium benefits" (a term with a pretty wide connotation) might well go forward with as little delay as may be. What is more important, responsible people show that they are not only thinking of this matter, but also beginning to move in it. Accordingly, builders and others interested have manifested some activity too. The medical journals lately contained announcements of a prize for a sanatorium architectural plan, and now a certain Scotch firm issue a descriptive circular with particulars of many inexpensive types of sanatorium accommodation. The semi-temporary constructions have many advantages too obvious to need recounting. The objection usually urged is that such erections need more keeping in repair than permanent ones. So undoubtedly they do, but we believe it has not been pointed out before that this is no disadvantage where pulmonary tuberculosis is in question. Far better sink less in prime cost and have more left for upkeep when that upkeep expenditure can go largely to benefit patients themselves. Outdoor painting is ideal work for a convalescent consumptive, and can be carried out very neatly by unskilled labour: there is no better form of "after care" than to take on two or three ex-patients to paint the place, finding them in board and lodging in well-designed huts or tents, and giving them five or ten shillings a week according as they are single or married. Just as the French *Goutte de Lait* system has shown that it is twice as effective to provide the nursing mother with a square meal, and let her nourish her offspring herself, as to preach to her the preparation of artificial foods for infants, so, surely, is finding healthy paid employment superior to the well-meant but rather hollow counsels of the dispensary doctor and the health visitor; and (with the present relative powers of nature and of art, of constitutional and of bacteriological therapeutics), so, in all probability, is it superior to ambulant tuberculin treatment. Recidivism is the great fault to be coped with in the sanatorium system, and after care, although quiet but persevering work is being done at one or two institutions, has not yet been paid anything like the attention it deserves, while as yet no review of Professor Pearson's last pamphlet has noticed his finding that Aberdeen, with no tuberculosis dispensaries at all, has experienced a more rapid drop in the tuberculosis death-rate than Edinburgh, which is, of course, the headquarters of that movement.

Another important publication at the present time is undoubtedly Dr. Marcus Paterson's book, and his timely warning may be emphasised that to dot the country with a great number of small sanatoria would be a very foolish business. The circular mentioned at the opening of these remarks has proposals even for ten-bed institutions, to be worked by a matron and a nurse. Besides the wastefulness of getting up house committees to look after dolls' houses like these, there is the fact that efficient sanatorium treatment presupposes unfailing obedience from patients. Now actually, as a matter of fact and not of compliment, there are very few women who can get this from working men who are not ill in bed but up and about; and those few will be found elsewhere engaged. The feminine genius is perfectly, naturally, intrinsically suited to nursing; hardly to managing directorship, on however small a scale. Dr. Paterson's book, with its occasional *naïvetés*, is quite interesting to read. In his short preface he claims originality for his work, and of one part of it this is certainly true. No one before him proved that consumptives could stand hard manual labour. Here he is generic to the first mariner and the first oyster swallower, and, to make no more fun, far superior to a dozen of conventionally distinguished, conventionally accepted medical hierarchs. But the auto-inoculation hare, however that may turn out in the pot, was none of his and Dr. Inman's starting. The *Erregung*, and more than that the idea and the inspiration were, as both would acknowledge, Sir Almroth Wright's, while the application to consumption, varying index, and nearly all of it, was worked out by Meakin and Wheeler and described in a paper in the *British Medical Journal*; worked out, moreover, on patients treated on ordinary Nordrach lines: so that it would seem that graduated labour is not essential to proper auto-inoculation. When Drs. Paterson and Inman subsequently wrote on the subject, they mentioned this article. But in this book of Dr. Paterson's it is not mentioned. If only for the compelling reason that Captain Meakin is now dead, the oversight should be corrected in another edition.

